# Non-travel related Hepatitis E virus genotype 3 infections in the Netherlands; A case series 2004 – 2006

**DOI:** 10.1186/1471-2334-8-61

**Published:** 2008-05-08

**Authors:** Katrine Borgen, Tineke Herremans, Erwin Duizer, Harry Vennema, Saskia Rutjes, Arnold Bosman, Ana Maria de Roda Husman, Marion Koopmans

**Affiliations:** 1 Centre for Infectious Disease Control, National Institute for Public Health and the Environment (RIVM), Antonie van Leeuwenhoeklaan 9, 3720 BA Bilthoven, The Netherlands; 2 European Programme for Intervention Epidemiology Training (EPIET), Tomtebodavägen 11A, SE-171 83 Stockholm, Sweden; 3 Preparedness & Response Unit, European Centre for Disease Prevention and Control (ECDC), Solna, Sweden

## Abstract

**Background:**

Human hepatitis E virus (HEV) infections are considered an emerging disease in industrialized countries. In the Netherlands, Hepatitis E virus (HEV) infections have been associated with travel to high-endemic countries. Non-travel related HEV of genotype 3 has been diagnosed occasionally since 2000. A high homology of HEV from humans and pigs suggests zoonotic transmission but direct molecular and epidemiological links have yet to be established. We conducted a descriptive case series to generate hypotheses about possible risk factors for non-travel related HEV infections and to map the genetic diversity of HEV.

**Methods:**

A case was defined as a person with HEV infection laboratory confirmed (positive HEV RT-PCR and/or HEV IgM) after 1 January 2004, without travel to a high-endemic country three months prior to onset of illness. For virus identification 148 bp of ORF2 was sequenced and compared with HEV from humans and pigs. We interviewed cases face to face using a structured questionnaire and collected information on clinical and medical history, food preferences, animal and water contact.

**Results:**

We interviewed 19 cases; 17 were male, median age 50 years (25–84 y), 12 lived in the North-East of the Netherlands and 11 had preexisting disease. Most common symptoms were dark urine (n = 16) and icterus (n = 15). Sixteen ate pork ≥ once/week and six owned dogs. Two cases had received blood transfusions in the incubation period. Seventeen cases were viremic (genotype 3 HEV), two had identical HEV sequences but no identified relation. For one case, HEV with identical sequence was identified from serum and surface water nearby his home.

**Conclusion:**

The results show that the modes of transmission of genotype-3 HEV infections in the Netherlands remains to be resolved and that host susceptibility may play an important role in development of disease.

## Background

Human hepatitis E virus (HEV) infections are increasingly recognized as an emerging disease in the industrialized countries [1]. HEV infections can be asymptomatic or symptomatic and cause acute hepatitis which usually is self-limiting but may progress into fulminate and even fatal disease [2]. In pregnant women a case-fatality rate up to 31% has been reported [3,4]. Persistent HEV infections have been described in two separate case reports [5,6]. HEV infections are caused by an RNA virus recently classified in the genus *Hepevirus *of the family *Hepeviridae *which contains four major recognized genotypes (1–4) infecting humans, with distinct geographical distribution [7]. Viruses belonging to a 5^th ^genotype have been identified in birds [8]. HEV is transmitted by the fecal-oral route and large waterborne outbreaks with high attack rate among young adults (15–40 years) have been described in developing countries with poor sanitary conditions [2,3,9,10]. In developed countries, HEV infections have primarily been related to travel to high-endemic regions. However, following the discovery of genotype 3 HEV in pigs and humans in the US in the mid 1990s, there is an increased recognition of these non-travel related HEV infections in industrialized countries. The HEV seroprevalence measured ranges from 0.9% in France to 2.6% in Italy [11], and recently a seroprevalence of 7.3% was measured among an adult population in Spain [12]. HEV infection is a non-notifiable disease in most countries and the national prevalence of infection and disease due to HEV is mostly unknown.

The long incubation period of HEV (15 – 60 days) complicates identification of a possible source and for most cases of sporadic infections the cause is unknown. In Japan, an identical viral sequence has been identified from a HEV patient and from leftovers of wild boar meat consumed by this patient [13]. This provided the first evidence for a direct link between animals and human infection and supports the hypothesis of HEV as a zoonotic agent. In addition, domesticated pigs and deer are thought to play a role in the transmission of HEV based on the high degree of sequence similarity between HEV strains from these animals and humans [14-18].

In the Netherlands, the first non-travel related case of HEV was diagnosed in 1990 followed by a 12 year period in which no such cases were identified [19]. In the late 1990s, HEV of genotype 3 was identified in 25/115 (22%) pooled fecal samples from Dutch pigs. The first cluster of non-travel related HEV infections in the Netherlands was described in three elderly patients and published in 2003 [20], followed by description of a fatal case in a 58 years old female with preexisting liver disease in 2004 [21]. No link or common source was identified in these investigations. Among 209 non-A, B, C hepatitis patients with no history of foreign travel, a HEV seroprevalence of 6% was found as opposed to 0.5% in a control group indicating locally acquired HEV infections in the Netherlands [22]. By genetic comparison of 14 pig strains with HEV strains from human cases a homology up to 97% was shown, suggesting that swine may be a reservoir of HEV [14]. A seroprevalence study among patients (n = 1027) with unexplained hepatitis with onset of illness between 1999 and 2003, found 9.8% seropositive (IgM and/or IgG) by ELISA confirmed by immunoblot. Of the patients with recent infection (IgM and IgG antibodies), more than 50% could be confirmed by virus detection in serum [18]. Since 2000, approximately 10 cases per year of non-travel related HEV infection have been diagnosed by the Dutch National Institute for Public Health and the Environment (RIVM) which most likely represents considerable underdiagnosis of HEV in the Netherlands.

We conducted a descriptive case study including environmental source tracing and molecular typing of strains to generate hypotheses about possible risk factors and transmission routes for non-travel related HEV infections in the Netherlands.

## Methods

### Patients

We performed active case finding by asking 99 medical microbiological laboratories, already collaborating with RIVM, for samples of patients with acute viral hepatitis who had tested negative for infection with hepatitis A, -B, -C, cytomegalovirus (CMV) and Epstein Barr virus (EBV). We defined a case as a person with laboratory confirmed HEV infection diagnosed after January 1^st ^2004, without travel to a high-endemic country in the three months prior to onset of illness. Laboratory confirmation was defined as a positive HEV RT-PCR result from either serum or stool sample and/or a positive IgM result combined with an IgG response after immunoblot confirmation performed as described by Herremans et al. [18]. For virus identification, a fragment of 148 bp of the gene encoding the major capsid protein (ORF2) was sequenced [18].

When a case was laboratory confirmed, permission from the microbiologist and the clinician was obtained before an appointment with the patient was made for the interview. This procedure is according to Dutch law and takes 1–2 months. The same investigator from RIVM together with local Municipal Health Service (MHS) staff interviewed all the cases face to face through a structured questionnaire collecting information on symptoms, medical history, food and water consumption, and contact with animals and surface water. Furthermore, in an open interview we asked about special events of possible relevance for identification of a source for the HEV infection (e.g. outdoor activities, hobbies, holidays, and cases' own suspicion). If informed consent was given, we collected serum samples from household contacts for serological HEV analysis. We entered and analyzed the data in Epi Info 3.3 and in MS Excel and performed geographical mapping of the cases by using ArcGis 9.1 (ESRI software).

### Environmental investigation

When a visit at the patient's home indicated a potential source of HEV in the close surroundings, we considered environmental investigation and if possible, performed sampling of foods, surface waters, sediments and animal droppings. Since surface waters were sampled months after onset of illness of the patients, sediment samples from these waters were taken as viruses from sewage discharge and wash-off of animal manure may accumulate there. Samples were processed and RNA extracted as previously published: surface water [23,24], sediment [25], liver [26] and animal feces [14,17]. HEV ORF2 RNA was detected by RT-PCR as previously described [14].

### Phylogenetic analyses

Sequencing and phylogenetic analyses were performed as described by van der Poel et al. [14]. The strains were compared with HEV sequences previously detected from humans and pigs in the Netherlands and with publicly available HEV sequences from other countries [27]. Alignment and clustering of the strains were done by Unweighted Pair Group Method with Arithmetic mean (UPGMA) analysis and minimal spanning tree in BioNumerics version 4.6 (Applied Maths, Sint-Martens-Latem, Belgium).

## Results

**Figure 1:**
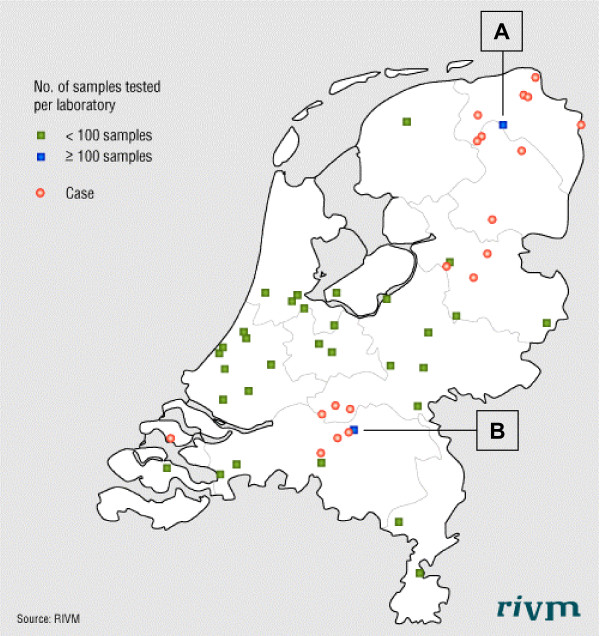
**Distribution of HEV cases and participating microbiological laboratories in the Netherlands**. Geographical distribution of 19 cases of non-travel related HEV and the 33 participating laboratories, the Netherlands 2004 – 2006. Laboratory A and B submitted 63% of the samples.

We received samples from 33 of the 99 laboratories (33%). From January 1 2004 till August 31 2006 we tested 748 samples from 694 patients for HEV. Two laboratories, A and B, submitted 277 and 193 samples, respectively, accounting for 63% of the total number of samples (Figure 1). The remaining 31 laboratories submitted between one and 68 samples (median: 3 samples) during the study period. A total of 19 cases met the case definition and were included in the study (Figure 1). Of these, 17 had a positive HEV PCR and two were confirmed by immunological method according to the case definition described in the Methods section. From laboratory A and B nine and five of the cases were identified, respectively. The remaining five cases were identified from two other laboratories.

Time of onset of disease for the 19 cases was from January 2004 to April 2006 (Table 1). The median time delay from onset of illness to the interview was 2.5 months (range 1 – 16.5). Twelve cases (66.7%) lived in the North-Eastern part of the country (Figure 1), 17 (89.5%) were male (sex ratio 17:2) and the median age was 50 years (range: 25–84). Nine cases were retired; five due to age and four due to various illnesses. None of the ten employed cases had a profession related to animals. Eleven cases (57.9%) reported preexisting disease; cardiovascular problems (n = 4), malignancies (n = 3), respiratory problems (n = 2) and other diseases (n = 3) (Table 1) and were under medication. Two of the cases with malignancies, aged 25 and 35, had received multiple blood transfusions within the three months before onset of illness. Eleven cases were hospitalized, with duration of stay from 2 – 23 days (median eight days). No case was fatal. The most common self reported symptoms were dark urine (n = 16) and icterus (n = 15). Fourteen cases (73.7%) reported fatigue lasting weeks to months after the acute illness (Table 2). For 13 cases a total of 18 contacts were serologically tested and all were negative for HEV IgM and IgG.

**Table 1:** Characteristics of 19 cases of non-travel related hepatitis E virus infections, the Netherlands 2004 – 2006.

**Case ID**	**Sex**	**Age**^a^	**Occupation**	**Preexisting disease**	**Duration of illness**	**Time delay**^b^
ID1	F	25	Policy worker	Malignancy	18 days	2.5
ID3	M	81	Retired	Cardiovascular, malignancy	>2 months	2
ID4	M	48	Businessman	None	17 days	2.5
ID5	M	58	Warehouse worker	None	~6 months	16.5
ID6	M	78	Retired	Respiratory	>2 months	2
ID7	M	45	Bricklayer	None	>2 months	2
ID8	M	35	Factory worker	Malignancy	-	2.5
ID9	M	50	Retired	Other	>1 month	1
ID10	M	57	Retired	Cardiovascular	~2 weeks	5
ID11	M	84	Retired	None	~2 weeks	2
ID12	M	36	Salesman	Respiratory	>2 months	5
ID13	M	38	Factory worker	None	~2 weeks	3
ID14	M	81	Retired	None	3 weeks	2
ID15	M	79	Retired	Cardiovascular	5 weeks	2
ID16	M	42	Retired	Other	-	4
ID17	F	50	Teacher	None	>2 months	2
ID18	M	34	Policy worker	None	3 weeks	3.5
ID19	M	57	Factory worker	Cardiovascular	3 weeks	3
ID22	M	54	Retired	Other	-	3.5

^a^Age at time of illness onset. ^b^Number of months from onset of illness to interview.

**Table 2:** Self-reported symptoms in 19 cases of non-travel related hepatitis E virus infections, the Netherlands 2004 – 2006.

**Symptom**	**Frequency**	**Percentage**	**Previously published^a ^(%)**
Icterus	15	78.9	~100
Nausea	5	26.3	29–100
Vomiting	3	15.8	29–100
Diarrhea	-	0	-
Abdominal pain	4	21.1	37–82
Decolorized stool	9	47.4	-
Dark urine	16	84.2	-
Pruritus	9	47.4	14–59
Malaise	14	73.7	~100
Headache	3	15.8	-
Fever	6	31.6	23–97

^a ^[[11]]

**Figure 2:**
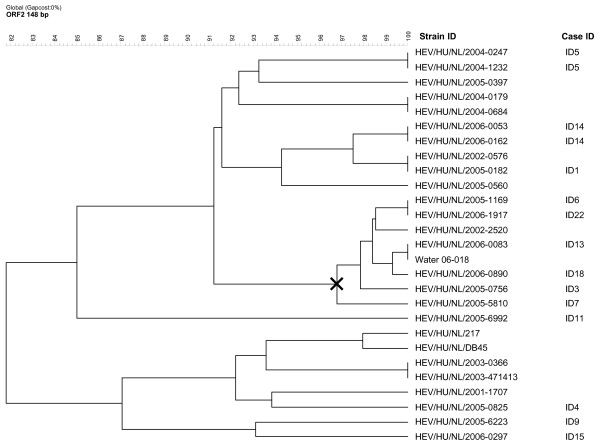
**Sequence homology between human and non-human HEV strains in the Netherlands**. Dendrogram showing genetic relatedness of 27 sequences of 148 nucleotides HEV RNA; 26 human strains and one environmental strain identified in the Netherlands. The strains from the 13 cases investigated from 2004–2006 are indicated by ID number. **X**: Cluster with more than 96% sequence homology. Accession numbers are shown in Table 5.

Among the 17 cases (89.5%) for whom the HEV RT-PCR was positive material was available from 13 cases for sequence analysis and phylogenic clustering of the 148 bp ORF2 RNA fragment. From one case (ID5) two HEV strains were available, one from serum and one from feces. All strains were identified as genotype 3. In the phylogenetic analysis of these strains together with 13 human HEV strains previously identified in the Netherlands, a 100% match was found for seven pairs of strains (Figure 2). Identical sequences were seen in the two strains from a serum and a fecal sample from the same patient (ID5) and in two cases with reported date of onset April 2005 (ID6) and April 2006 (ID22) with no identified epidemiological link. Furthermore, for two cases with date of onset December 2004 (ID1) and February 2002 who lived in the North and the South of the country, respectively, the sequences were identical. The case from 2002 was not included in our study and no further information could be obtained. Eight strains, seven from cases and one from a surface water sample, formed a cluster with more than 96% sequence homology (Figure 2). Six of these strains came from recent cases and three of these cases lived in the same Dutch province (Overijssel). No other cases were identified from this province.

**Figure 3:**
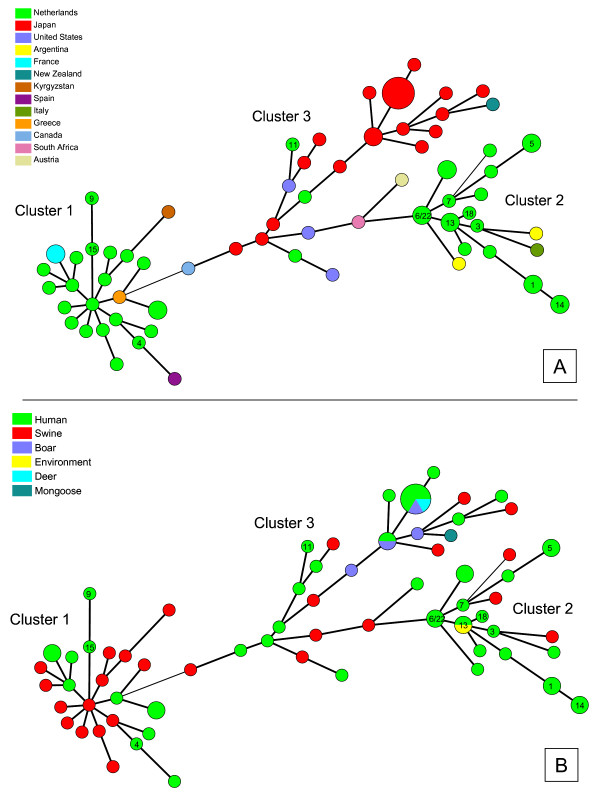
**Genetic relatedness between HEV strains from human and non-human sources and from various countries**. Minimal spanning trees of 81 sequences of 148 nucleotides HEV RNA showing genetic distances between genotype 3 HEV strains from humans and animals. **A**: strains labeled by geographical origin. **B**: strains labeled by biological origin. The 13 recent Dutch cases are marked with ID numbers inside the colored circles.

We extended the cluster analyses to 81 overlapping sequences, including internationally available genogroup 3 HEV strains from animals and humans [27]. Twelve of the 13 HEV strains from patients in this study segregated into two main sub lineages within genotype 3 HEV (Figure 3). Each of these sub lineages contains strains collected from surveillance in pigs in the Netherlands and strains from symptomatic cases in the Netherlands. Nine of the strains clustered with strains mainly from humans and three strains clustered with strains mainly from pigs (Figure 3B). One strain (ID11) clustered with strains outside the two main sub lineages.

Sporadic contact with dogs was reported by 14/19 (73.7%) cases of which six (31.5%) had their own dog. From the dog of one patient, a fecal sample was collected and analyzed but no positive signal for HEV RNA was detected by RT-PCR. None of the cases had contact with rats although eight (42.1%) had contact with other rodents (rabbits and guinea pigs) (Table 4). From the rabbit of one patient a fecal sample was collected and analyzed but no HEV RNA was detected. Analyses of fecal samples from cows (n = 16), horses (n = 4), ponies (n = 2), an Indonesian pig (n = 1), and one pooled cow/sheep fecal sample did not show the presence of HEV RNA. Pig manure used in the garden by one patient did also not reveal the presence of HEV RNA.

Sixteen cases (84.2%) reported eating pork more than once a week (Table 3). None of the cases ate raw pork or raw organs from any animals, although nine (47.3%) and three (15.8%) cases did consume heat processed pork and chicken liver, respectively, at a frequency less than once a week. One case had pork liver stored in the freezer available for analysis but no HEV RNA could be detected. Eight cases (42.1%) reported eating dried sausages which traditionally are made of uncooked pork. One case had consumed dried fruits and nuts wrapped in banana leaf from Curacao which also tested negative for HEV RNA. Daily consumption of tap water and mineral water was reported by 16 and three cases, respectively. Tap water at the time of probable exposure was not available for sampling and no mineral waters were left for analyses.

**Table 3:** Food consumption for 19 cases of non-travel related hepatitis E virus infections, the Netherlands 2004 – 2006.

	**≥ 1/week**		**<1/week**		**Never**	
			
**Food product**	**n**	**%**	**n**	**%**	**n**	**%**
Pork	16	84	3	16	0	-
Beef	9	47	9	47	1	5
Chicken	13	68	6	32	0	-
Fish	9	47	9	47	1	5
Mutton	1	5	2	11	16	84
Turkey	1	5	9	47	9	47
Shellfish	1	5	6	32	12	63
Raw milk	1	5	2	11	16	84
Game/wild	0	-	5	26	14	74
Organs	0	-	7	37	12	63

**Table 4:** Animal contact in 19 cases of non-travel related hepatitis E virus infections, the Netherlands 2004 – 2006.

**Animal**	**Number of cases**	**Percentage**
Rats	0	0
Rodents other than rats	7	36.8
Pigs	1	5.3
Deer	2	10.5
Wild boar	0	0
Hare	1	5.3
Sheep	3	15.8
Goat	4	21.1
Cattle	6	31.6
Horse	7	36.8
Poultry	2	10.5
Dog	14	73.7
Cat	6	31.6

Five of the cases (26.3%) had at least one incident of contact with surface water during the incubation period. None of the six sediment samples collected in surface waters nearby two patients' homes tested positive for the presence of HEV RNA. Ditch water near another patent's home tested negative for HEV RNA. For one case (ID13), three surface water samples were taken three months after onset of illness; the canal water used for swimming and the pond in the garden both tested negative. However, a sample taken from a ditch near the house yielded a positive HEV RNA signal. This ditch received sewage effluent from a leakage in the household septic tank. Sequencing analyses revealed a 100% match between the sequence obtained from the PCR products amplified from the serum sample of the HEV patient and the ditch water (Figure 2 and Figure 3B). Accession numbers are shown in Table 5.

**Table 5:** Accession numbers.

**Accession number**	**Strain ID**	**Case ID**
AB385842	HEV/HU/NL/2002-2520	
AB385843	HEV/HU/NL/2004-0247	ID5
AB385844	HEV/HU/NL/2005-5810	ID7
AB385845	HEV/HU/NL/2005-6223	ID9
AB385846	HEV/HU/NL/2005-6992	ID11
AB385847	HEV/HU/NL/2006-0053	ID14
AB385848	HEV/HU/NL/2006-0083	ID13
AB385849	HEV/HU/NL/2006-0162	ID14
AB385850	HEV/HU/NL/2006-0297	ID15
AB385851	HEV/HU/NL/2006-0890	ID18
AB385852	HEV/HU/NL/2006-1917	ID22
DQ200273	HEV/HU/NL/217	
DQ200274	HEV/HU/NL/2001-1707	
DQ200275	HEV/HU/NL/2002-0576	
DQ200277	HEV/HU/NL/2003-0366	
DQ200278	HEV/HU/NL/2003-471413	
DQ200279	HEV/HU/NL/2004-0179	
DQ200280	HEV/HU/NL/2004-0684	
DQ200282	HEV/HU/NL/2004-1232	ID5
DQ200283	HEV/HU/NL/2005-0182	ID1
DQ200284	HEV/HU/NL/2005-0397	
DQ200287	HEV/HU/NL/2005-0560	
DQ200289	HEV/HU/NL/2005-0756	ID3
DQ200292	HEV/HU/NL/2005-0825	ID4
DQ200293	HEV/HU/NL/2005-1169	ID6
DQ200295	HEV/HU/NL/DB45	

## Discussion

We identified 19 cases with non-travel related HEV infection with onset of illness from January 2004 to April 2006. This supports the hypothesis that HEV is endemic in the Netherlands, although infections through consumption of contaminated imported foods can not be ruled out. The geographical distribution of the cases is striking, with the majority of cases living in rural areas in the North/Eastern part of the country and a cluster of cases in the South. However, these results correspond to the location of the two laboratories submitting the majority of samples and must be interpreted with caution. It might reflect a reporting bias as diagnostics of HEV in non-travelers depend on the alertness of the clinician and the microbiologist in charge. The first cases of non-travel related HEV in the Netherlands were reported in the North [20]. In a study from England and Wales, living in coastal or estuarine areas was reported as a risk factor for acquiring non-travel-associated HEV disease [28].

The high male:female ratio and the relatively high median age is difficult to explain but still consistent with previously published data [1,2,28,29]. More than half of the cases had one or more preexisting diseases for which they received medical treatment. This might have influenced the testing scheme of the clinicians and created a selection bias towards a more vulnerable/susceptible case population. However, recent reports describe association between HEV infection and pre-existing disease, such as hyperthyroidism [30] and chronic liver disease [31]. In this case series none of the cases reported other liver disease but their medication might render the liver more susceptible to infection. Sub-clinical infections of HEV are well known [2] and the immune system of an individual might play an important role in the development from infection to clinical disease. Three cases were immunocompromised due to malignant disease and one due to usage of immunosuppressive drugs. Two of the cases with malignancies had received multiple blood transfusions during the estimated incubation period which might be the source of their infection, as transmission of HEV via blood has previously been described [32-34]. Trace back of donor blood for these two cases was, however, not considered at the time of this investigation because a relationship with blood donation was thought to be unlikely by the treating physician. Blood donors in the Dutch blood bank have not previously been screened for HEV but the potential for blood borne HEV has now been included in the risk analysis of an advisory committee of the Dutch blood bank. In a study by Zaaijer et al in 1995, 0.4% of 1,275 samples from blood donors tested positive for HEV antibodies [35]. In a study of sera from 167 healthy individuals two samples (1.2%) were IgG and IgM positive by ELISA and 2/65 (3.1%) by immunoblot [36] although the seroprevalence of HEV in the general Dutch population remains unknown.

The genotyping results of the HEV strains from the PCR positive cases further support the hypothesis of an indigenous HEV reservoir in the Netherlands although we have no evidence for direct zoonotic transmission. There was one 100% match between a case and a surface water sample taken in his close surroundings, but this likely reflects shedding into the environment from this patient. Remarkably, however, this water sample was taken almost three months after onset of illness in the patient. As sewage from the household septic tank was let out in this ditch, the patient might have contaminated the water with HEV. HEV is known to be stable in environmental samples, particularly in watery environments and has previously been identified in sewage samples [37]. As part of a routine control of water from the large river Maas in the Netherlands HEV was isolated from 2/12 samples analyzed in September 2004 and March 2005 [38].

Our epidemiological data do not directly support the "swine as HEV reservoir hypothesis", as only one of the cases had direct contact with pigs in the estimated incubation period and none of the cases reported eating raw pork or raw organs from pork. Dried sausages might, however, serve as a proxy for consumption of pork that is insufficiently processed for HEV elimination and should be an issue for further investigation. In 2005, HEV was isolated from raw pork liver obtained from a butchers shop in the Netherlands [26], showing that people can be exposed through handling or consumption of raw pork livers also in our country. In general, pork consumption was high among the cases, with 78.9% eating pork (well done) more than once a week. Comparable data for the general population is not available, although pork is estimated to represent approximately 25% of the total amount of meat purchased on household level in the Netherlands.

Contact with dog and rodents other than rats might represent a possible riskfactor to be further investigated. The cases who were owners of dogs reported feeding their dogs only canned and dried feed, no raw meat or organs. Due to the long incubation period and time delay from onset of illness to the interview took place (Table 1) we collected data on food consumption using a food preference list and on contact with animals in general and not directly related in time to the HEV infection. This might create a recall bias and specific incidents with possible importance for the transmission of HEV occurring in the incubation period might be left out.

We performed a limited contact investigation around a selection of the cases depending on the household composition and the consent given by the household members. However, none of the 18 serum samples tested showed any indications of a HEV infection, confirming the previously published findings from high endemic areas that HEV has limited person to person transmission [2].

## Conclusion

Our study shows that sporadic cases of non-travel related HEV infections continue to occur in the Netherlands and that this most likely is caused by endemic HEV genotype-3 strains. Despite an extended trawling questionnaire combined with environmental investigations and molecular characterization of strains we were not able to point out any common source for the infections in these 19 cases. A direct zoonotic transmission of HEV from pigs to humans could not be confirmed, although food borne transmission via inappropriately processed pork might have occurred. As for most of the cases various possible sources of HEV were reported, an indirect route of transmission is the most likely explanation for these infections. Individual factors as gender, age and pre-existing disease may play an important role in development of disease after infection with HEV genotype 3. How often HEV infections do occur in the Netherlands should be investigated in a seroprevalence study of the general Dutch population.

## Competing interests

The authors declare that they have no competing interests.

## Authors' contributions

KB participated in the design of the study including development of the questionnaire, interviewed all the cases, managed and analyzed the epidemiological data and drafted the manuscript. TH participated in the design of the study, managed the human sample collection, carried out the immunoassays and PCR of human samples and participated in drafting the manuscript. ED participated in the design of the study, in the sequence alignment and in drafting the manuscript. HV carried out the molecular genetic studies of the human strains and the sequence alignment of human and non-human strains. SR carried out the molecular genetic studies of the non-human strains and participated in drafting the manuscript. MK, AB, AMRH conceived of the study, participated in its design and coordination and in drafting of the manuscript. All authors read and approved the final manuscript.

## Pre-publication history

The pre-publication history for this paper can be accessed here:


